# 

**Published:** 1861-02

**Authors:** 


New Series, Vol. 4. No. 2. Whole Series, Vol. 18, No. 2.

### THE CHICAGO

### MEDICAL JOURNAL.

DANIEL BRAINARD, M. D.,
J. ADAMS ALLEN, M. D.,
Editors and Proprietors.

FEBRUARY, 1861.

CHICAGO:
WM. PIGOTT, BOOK AND JOB PRINTER, 126 CLARK STREET.

1861.

